# Cardiac Depression in Pigs after Multiple Trauma – Characterization of Posttraumatic Structural and Functional Alterations

**DOI:** 10.1038/s41598-017-18088-1

**Published:** 2017-12-19

**Authors:** M. Kalbitz, S. Schwarz, B. Weber, B. Bosch, J. Pressmar, F. M. Hoenes, C. K. Braun, K Horst, T. P. Simon, R. Pfeifer, P. Störmann, H Hummler, F. Gebhard, H. C. Pape, M. Huber-Lang, F. Hildebrand, TREAT Research Group, B. Auner, B. Relja, I. Marzi, G. Marx, A. Haug, L. Egerer, M. v. Griensven, R. Tolba, K. Reiss, S. Uhlig, M. Teuben, K. Almahmoud, Y. Kalbas, H. Lüken, K. Almahmoud

**Affiliations:** 1 Department of Traumatology, Hand-, Plastic-, and Reconstructive Surgery, Center of Surgery, University of Ulm, Ulm, Germany; 2 Division of Neonatology and Pediatric Critical Care, University of Ulm, Ulm, Germany; 3 Institute of Clinical and Experimental Trauma-Immunology, University of Ulm, Ulm, Germany; 4 Department of Orthopaedic Trauma, RWTH Aachen University, Aachen, Germany; 5 Department of Intensive Care and Intermediate Care, RWTH Aachen University, Aachen, Germany; 6 Department of Trauma Surgery, University Hospital Zurich, Zurich, Switzerland; 7 Department of Trauma-, Hand- and Reconstructive Surgery, University Hospital Frankfurt, Goethe University, Frankfurt/Main, Germany; 8 Department of Trauma Surgery, Technical University Munich, Munich, Germany; 9 Institute for Laboratory Animal Science and Experimental Surgery, RWTH Aachen University, Aachen, Germany; 10 Institute of Pharmacology and Toxicology, RWTH Aachen University, Aachen, Germany; 11 Harald Tscherne Research Laboratory, RWTH Aachen University, Aachen, Germany

## Abstract

The purpose of this study was to define the relationship between cardiac depression and morphological and immunological alterations in cardiac tissue after multiple trauma. However, the mechanistic basis of depressed cardiac function after trauma is still elusive. In a porcine polytrauma model including blunt chest trauma, liver laceration, femur fracture and haemorrhage serial trans-thoracic echocardiography was performed and correlated with cellular cardiac injury as well as with the occurrence of extracellular histones in serum. Postmortem analysis of heart tissue was performed 72 h after trauma. Ejection fraction and shortening fraction of the left ventricle were significantly impaired between 4 and 27 h after trauma. H-FABP, troponin I and extracellular histones were elevated early after trauma and returned to baseline after 24 and 48 h, respectively. Furthermore, increased nitrotyrosine and Il-1β generation and apoptosis were identified in cardiac tissue after trauma. Main structural findings revealed alteration of connexin 43 (Cx43) and co-translocation of Cx43 and zonula occludens 1 to the cytosol, reduction of α-actinin and increase of desmin in cardiomyocytes after trauma. The cellular and subcellular events demonstrated in this report may for the first time explain molecular mechanisms associated with cardiac dysfunction after multiple trauma.

## Introduction

In the United States approximately 30,000 patients with blunt cardiac trauma were recorded per year^1^. Heart injury was identified as an independent predictor for poor outcome^2^ and was associated with prolonged ventilation interval^3^ and longer hospital stay^4^. The clinical feature of the so called “commotio cordis” is associated with dysrhythmias, including ventricular fibrillation and sudden cardiac arrest^5,6^. In patients with hypotension disproportionate to the estimated blood loss and inadequate response to fluid resuscitation cardiac injury should be considered^7^. Accordingly, myocardial contusion correlates well with the incidence of perioperative hemodynamic instability^8^ and cardiac complications^9^.

Pathophysiological effects of blunt cardiac injury such as firmness of cardiac tissue was found in a pig model of blunt chest trauma^10^. In an isolated Langendorff perfused rat heart model early onset of specific impairment of the left ventricle such as an increase of left ventricular end-diastolic pressure occurred after cardiac contusion and was associated with increased cardiac specific troponin levels immediately after trauma^11^. Furthermore, a direct relationship between serum troponin levels and survival of trauma patients was identified^12^.

Nevertheless, following tissue damage endogenous alarmins interact with receptors for proinflammatory immune activation^13^. After major trauma in humans a series of circulating biomarkers such as IL-6^14^ and complement anaphylatoxines including C5a and C3a^15^ were elevated. Activation of the serine protease system after severe tissue trauma further amplifies cleavage of C5 and C3, generating functional C5a and C3a^16^, respectively. Of note, C5a has been found to induce dramatic contractile dysfunction in cardiomycytes *in vitro* by interaction with C5aR1^17^ and *in vivo* during sepsis. DAMPs such as circulating nucleosomes have been reported to correlate with the injury severity score in humans and were broken down into individual histones^18^, interacting with a variety of cells including cardiomyocytes presumably via toll like receptors^19^. During systemic inflammation release of histones has been linked to organ failure and lethality^18,19^.

After multiple trauma the triad of hypothermia, acidosis and coagulopathy is associated with high mortality rates^20^. Early rewarming, hemostasis, fluid and blood replacement are crucial to improve patient outcome and to prevent multiple organ dysfunction (MODS). After multiple trauma patients faces a second hit, for example surgical intervention, which in the state of vulnerable condition is associated with hyperinflammation and multiple organ dysfunction (MODS). Early total care concept including femoral nailing is associated with a more extensive surgical intervention compared to damage control concept, where surgical stabilization of fractures is performed minimally invasive by external fixation. A significant reduction of systemic inflammation in patients was observed in DCO compared to ETC^21^.

However, the mechanistic basis of depressed cardiac function after trauma is still elusive. Therefore, in the present study a large animal model of polytrauma in pigs including blunt chest trauma, femur fracture, liver laceration followed by pressure-controlled haemorrhage was applied to investigate morphological, immunological and functional changes of cardiac injury early after multiple trauma.

## Results

### Cardiac dysfunction and appearance of extracellular histones in serum after multiple trauma

**Figure 1:**
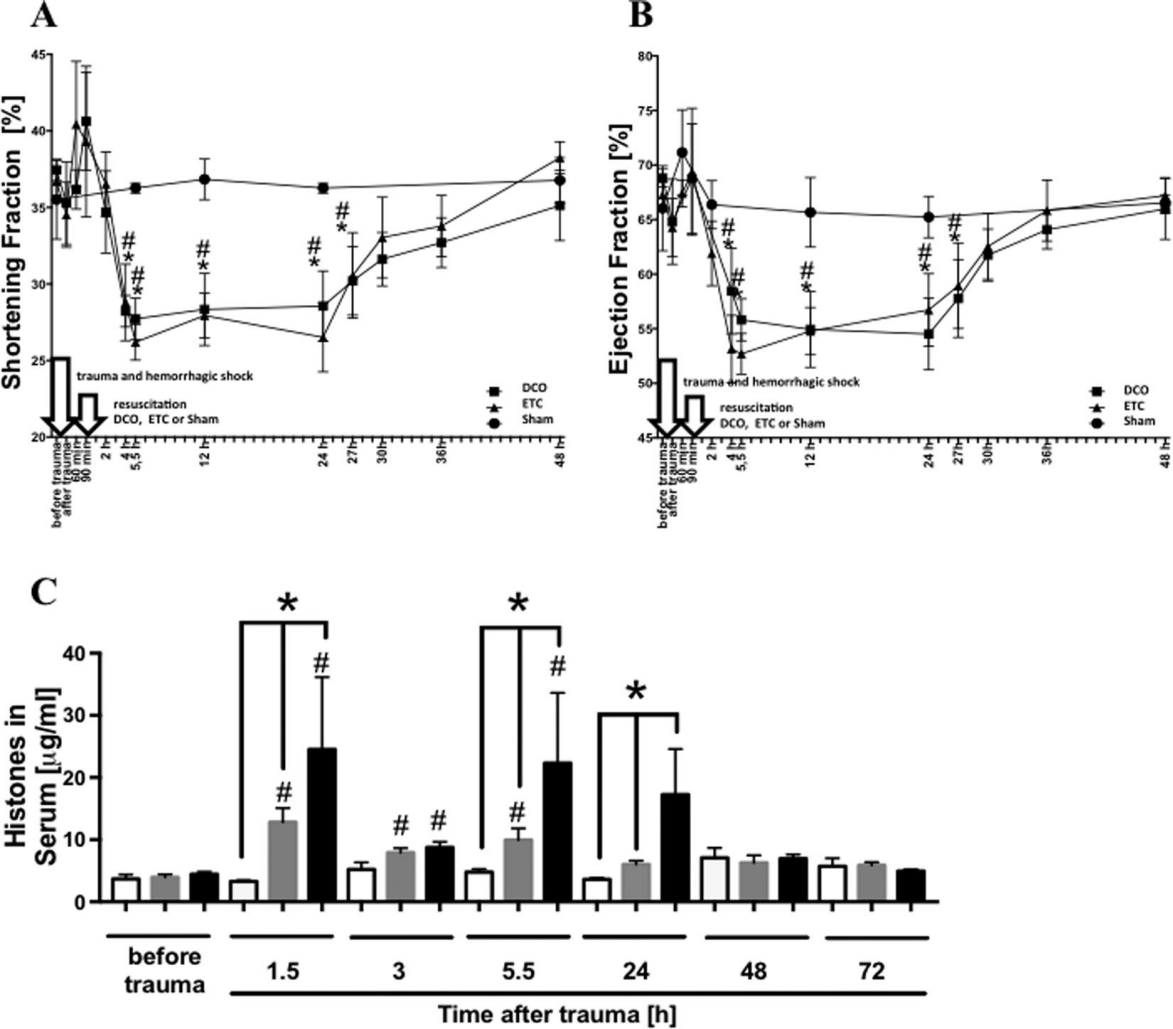
Hemodynamics and extracellular histones in serum. (**A**) Shortening fraction (SF%) of left ventricle in sham, multiple trauma followed by damage control orthopedics (DCO) or multiple trauma followed by early total care orthopedics (ETC) at indicated time points. (**B**) Ejection fraction (EF%) of left ventricle in sham, multiple trauma followed by damage control orthopedics (DCO) or multiple trauma followed by early total care orthopedics (ETC) at indicated time after trauma. n = 6 pigs in each group, ^#^DCO p < 0.05. *ETC p < 0.05. (**C**) Time course for extracellular histone appearance in serum from pigs after multipe trauma and DCO treatment (grey bars), ETC treatment (black bars) or sham (white bars), *differences to sham procedure were significant, p < 0.05; ^#^differences to baseline concentrations were significant, p < 0.05. For all frames n = 6 for each bar.

(Figure 1) To determine whether functional defects in cardiovascular performance occur after multiple trauma, echocardiographic parameters were assessed before, directly as well as 1, 1.5, 2, 4, 5.5, 12, 24, 27, 30, 36 and 48 h after trauma. Pigs subjected to multiple trauma demonstrated significant abnormalities in systolic parameters such as FS and EF (Fig. 1) in both treatment groups, DCO and ETC. FS (frame A) and EF (frame B) were reduced between 4 h and 27 h after trauma, respectively, compared to baseline whereas in sham treated animals both parameters remained similar to baseline measurements. Extracellular histone concentration in serum was elevated 1.5, 3 and 5.5 h after trauma compared to baseline in both treatment groups DCO and ETC and 1.5, 5.5 and 24 h compared to sham procedure (frame C).

### Elevated H-FABP and troponin I serum concentration and extravasation of erythrocytes in left ventricular tissue after multiple trauma

**Figure 2:**
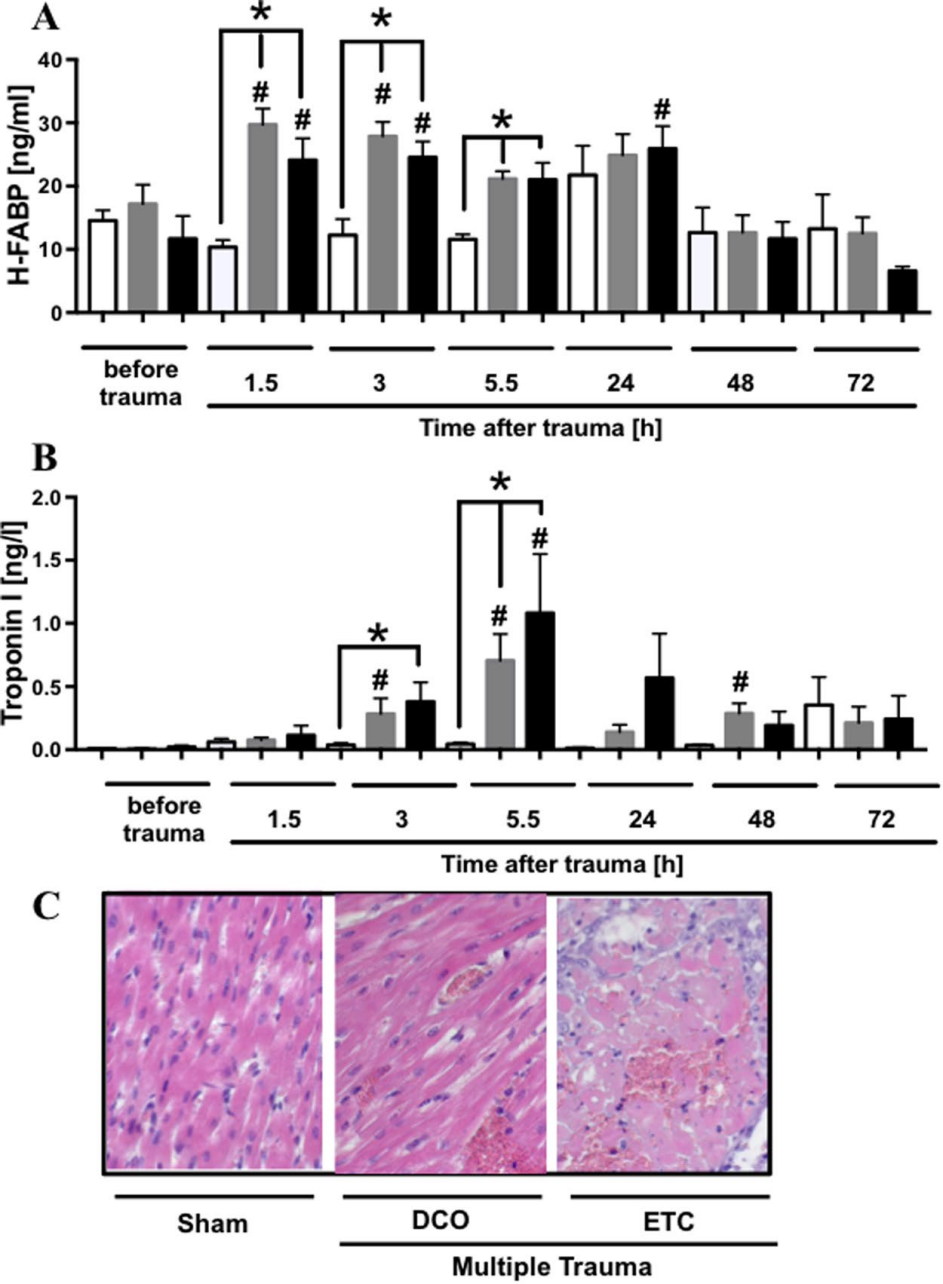
Cardiac injury after multiple trauma assessed by cardiac specific serum markers. (**A**) Time course of appearance heart-type fatty acid binding protein (H-FABP) after multiple trauma and damage control treatment (DCO, grey bars) or early total care treatment (ETC, black bars) or sham procedure (white bars) in pigs. (**B**) Time course of cardiac specific troponin I in serum after multiple trauma and damage control treatment (DCO, grey bars) or early total care treatment (ETC, black bars) or sham procedure (white bars). p < 0.05; *differences were significant to sham procedure, ^#^differences were significant to baseline. For all frames n = 6 for each bar. H.E. staining of left ventricle in sham (left), after multiple trauma and DCO (middle) or ETC (right) treatment, respectively (frame **C**).

(Figure 2) To estimate cardiac injury after multiple trauma in pigs, serum markers specific for cardiac cell damage were assessed at different time points after trauma (Fig. 2). H-FABP serum concentration was elevated as early as 1.5 h after trauma and after 3 and 5.5 h compared to sham procedure and 1.5 h and 3 h compared to baseline after trauma in both treatment groups (DCO and ETC) and after 24 h in ETC treatment group compared to baseline. Cardiac specific troponin I serum concentrations in pigs were assessed before and at indicated time points after multiple trauma. Troponin I concentrations in serum were elevated after trauma in both treatment groups (DCO and ETC) 3 and 5.5 h after trauma compared to baseline and compared to sham procedure as well as 48 h after trauma in DCO treated animals compared to baseline. Furthermore, H.E. staining of left ventricle tissue revealed no extravasation of erythrocytes in sham animals (0/6) whereas in animals objected to multiple trauma with DCO (2/6) or ETC (5/6) treatment extravasation of erythrocytes was present.

### Local inflammation in cardiac tissue after multiple trauma

**Figure 3:**
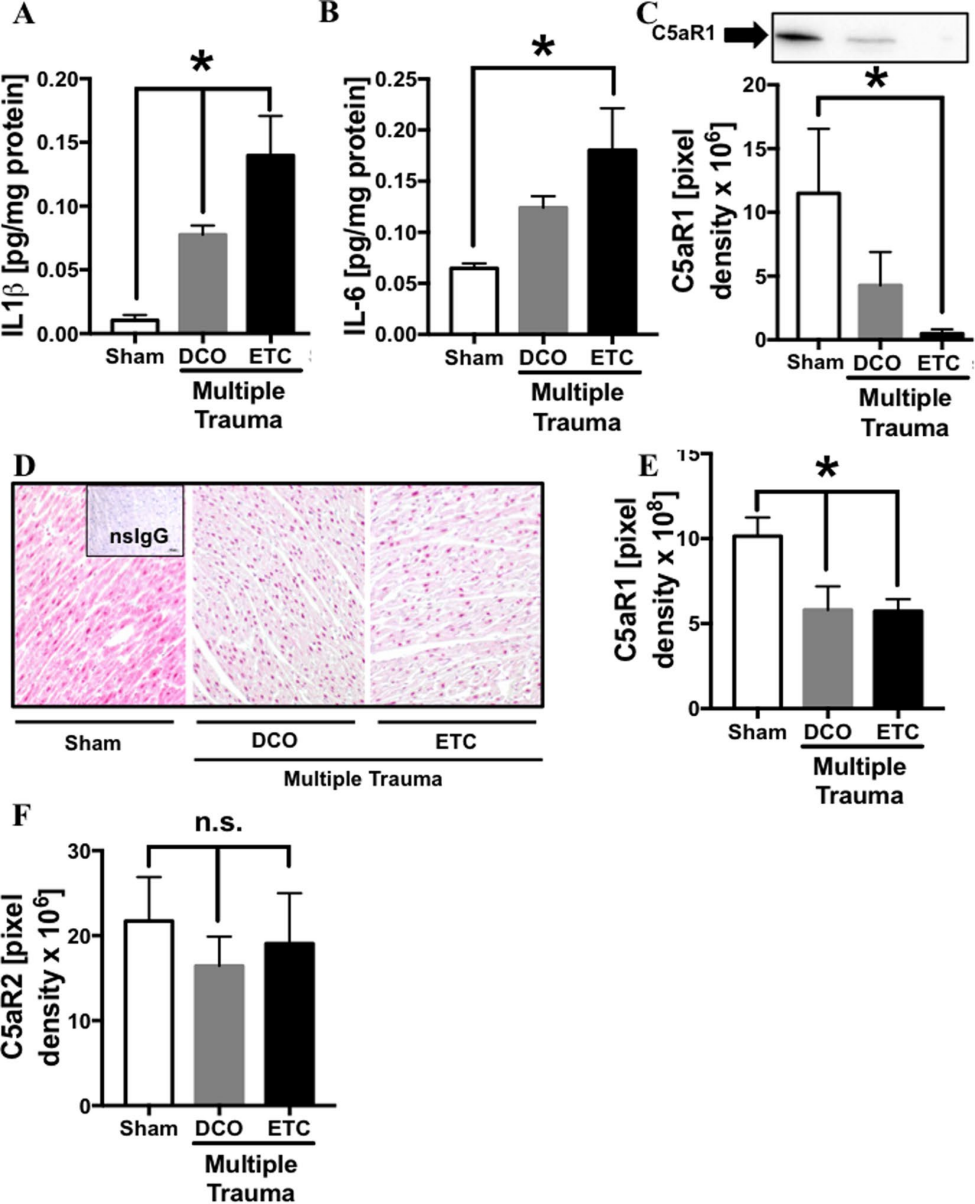
Local inflammation in cardiac left ventricular tissue 72 h after multiple trauma or sham procedure. (**A**) Elevation of proinflammatory cardiodepressive cytokine IL-1β concentration in left ventricular homogenates after multiple trauma in both treatment groups DCO (grey bar) and ETC (black bar) compared to animals after sham procedure (white bar) assessed by ELISA. (**B**) Elevation of proinflammatory cardiodepressive cytokine IL-6 concentration in left ventricular homogenates after multiple trauma in ETC (black bar) treatment group compared to animals after sham procedure (white bar) assessed by ELISA. (**C**) Representative western blot of C5aR1 in left ventricular homogenates in pigs after trauma and DCO (grey bar) or ETC (black bar) treatment and in sham treated animals (white bar), pixel densitry was assessed for quantification. Representitive C5aR1 staining of left ventricle tissue after trauma and DCO (middle) or ETC (right) treatment and in sham (left) treated animals (**D**). (**E**) Changes in density of C5aR1 staining in left ventricular tissue after trauma and DCO (grey bar) or ETC (black bar) treatment and in sham treated animals (white bar). (**F**) Changes in C5aR2 protein expression in left ventricular tissue after trauma and DCO (grey bar) or ETC (black bar) treatment and in sham treated animals (white bar). For each bar n = 6 samples, *p < 0.05.

(Figure 3) Homogenates and tissue sections of the left ventricle were obtained 72 h after multiple trauma followed by DCO or ETC treatment or after sham procedure, respectively. IL-1β concentration of sham treated animals was low whereas after multiple trauma in both treatment groups, DCO and ETC, IL-1β concentrations were significantly elevated (frame A). IL-6 concentration was elevated after multiple trauma/ETC (frame B) compared to sham treated animals. Western Blot analysis revealed reduction of C5aR1 protein in left ventricular tissue after multiple trauma/ETC treatment compared to sham treated animals (frame C). As assessed by immunhistochemistry in left ventricle sections after multiple trauma and either DCO or ETC treatment (frame D) diminished C5aR1 staining was identified compared to sham procedure (frame E) whereas complement receptor C5aR2 protein measurement in left ventricular tissue failed to show any differences between sham treated animals and pigs after multiple trauma (frame F).

### Translocation of Cx43 with co-translocation of ZO-1

**Figure 4:**
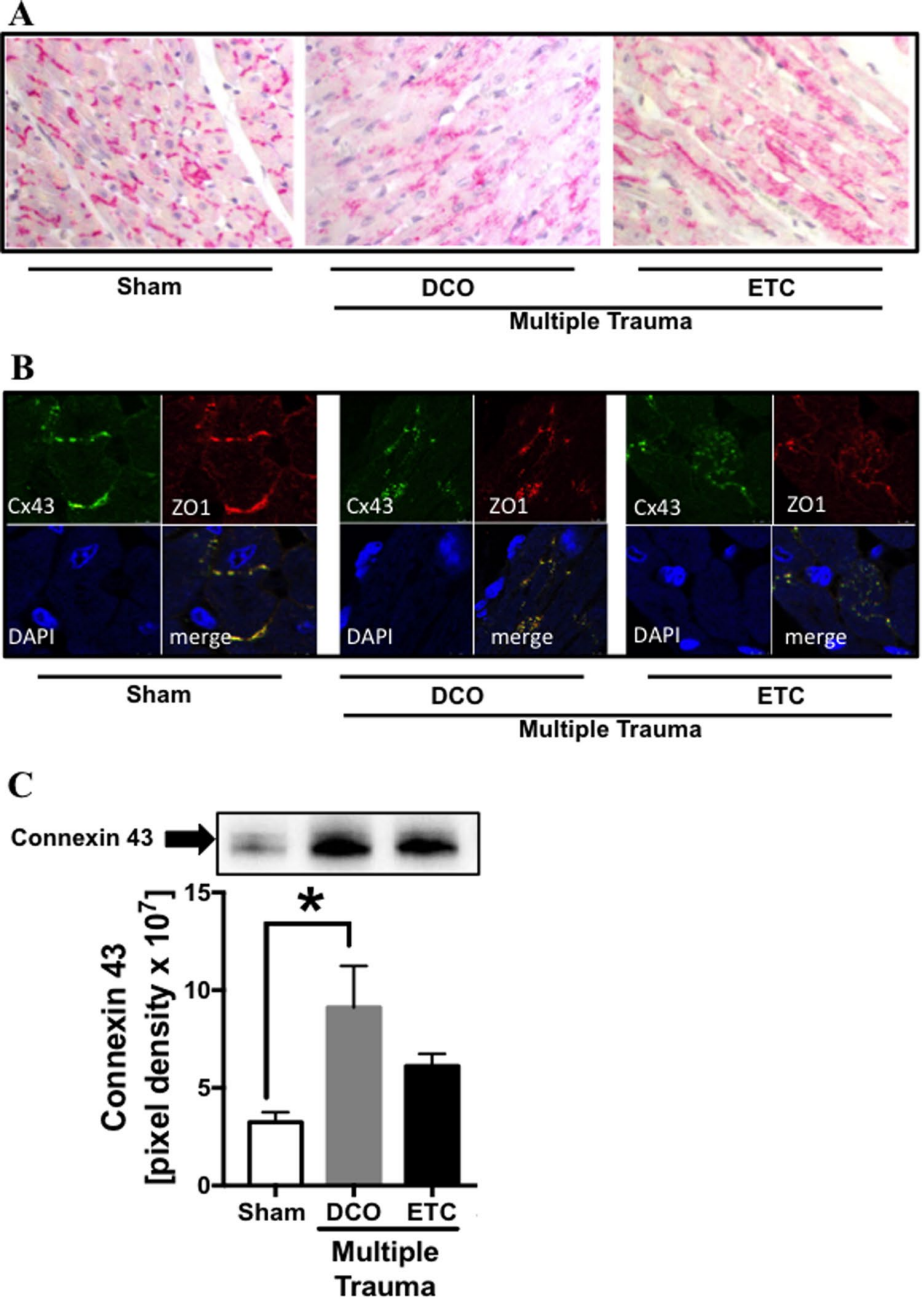
Alteration of gap junctional proteins after multiple trauma. (**A**) Representative distribution of connexin 43 (Cx43) in cardiac tissue in pigs after sham (left) procedure or after multiple trauma followed by DCO (middle) or ETC (right) treatment. (**B**) Representative immunfluorescence staining and confocal imaging of Cx43 (green) and ZO-1 (red), blue staining of nuclei with DAPI, in cardiac tissue of pigs after sham procedure (left) or after multiple trauma followed by DCO (middle) or ETC (right) treatment. (**C**) Representative western blot of Cx43 in left ventricular homogenates of sham treated pigs (white bar) or pigs after DCO (grey bar) or ETC (black bar). *p < 0.05, n = 6 each bar.

(Figure 4) To determine whether gap junction proteins in the heart were altered after multiple trauma immunohistochemistry staining of Cx43 gap junction protein in left ventricular tissue sections was performed. In sham treated animals Cx43 was located in intercalated discs whereas after trauma followed by DCO or ETC Cx43 was translocated and scattered into the cytosol (frame A). Subsequently Cx43 and ZO-1 (frame B) were co-translocated into the cytosol compared to sham procedure in both treatment groups DCO and ETC. Western blot analysis revealed increased Cx43 protein concentrations in left ventricular homogenates after multiple trauma and DCO treatment (frame C).

### Opposing trend of α-actinin and desmin expression in cardiac tissue after trauma

**Figure 5:**
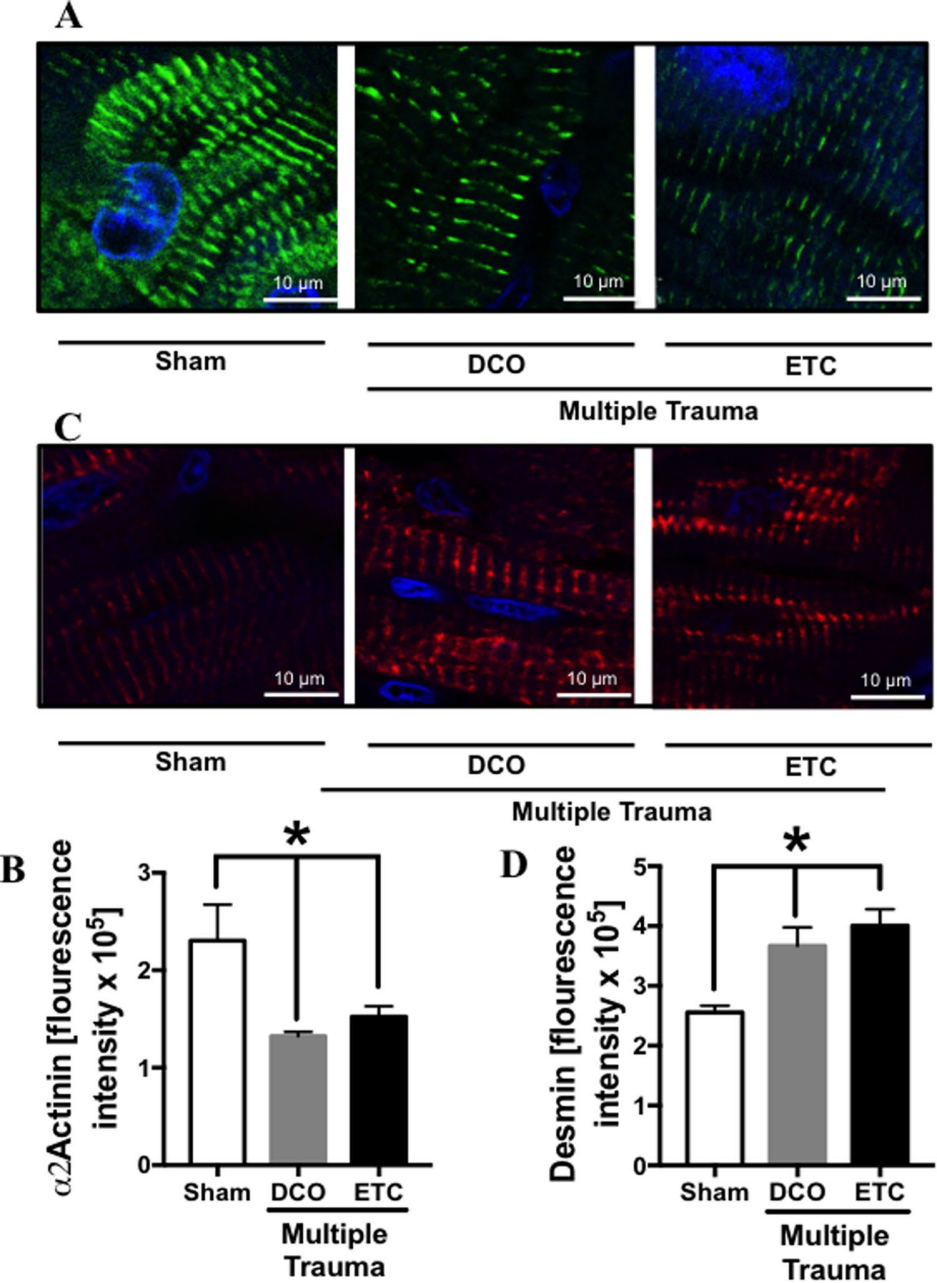
Representative confocal imaging of α2-actinin (**A**) and desmin (**C**) in pig left ventricle tissue sections. Blue staining of nuclei with DAPI, α2-actinin labeled with Alexa Flour® 488 (green), desmin labeled with PE (red). α2-actinin staining in left ventricles of sham treated animals (left) or 72 h after multiple trauma and DCO (middle) or ETC (right) treatment. Flourescence intensity of α2-actinin staining and confocal imaging in sham (white bar), multiple trauma and ETC treatment (grey bar) or ETC treatment (black bar) (**B**). Desmin staining in left ventricles of sham (left) treated animals or 72 h after multiple trauma and DCO (middle) or ETC treatment (right). Flourescence intensity of desmin staining and confocal imaging in sham (white bar), multiple trauma and ETC treatment (grey bar) or ETC treatment (black bar) (**D**). Scale bar represents 10μm. Representation of flourescence intensity in arbitrary units (AU) (**D**), n = 6, p < 0.05.

(Figure 5)Whereas expression of α-actinin in cardiomyocytes was reduced after multiple trauma followed by DCO and ETC compared to sham procedure (frame A), which was confirmed by fluorescence intensity measurement (frame B), expression of desmin was increased (frame C), likewise confirmed by fluorescence intensity measurement (frame D).

### Trauma-induced nitrosative stress in cardiac tissue in both treatment groups and increased caspase 3 expression in DCO trauma group

**Figure 6:**
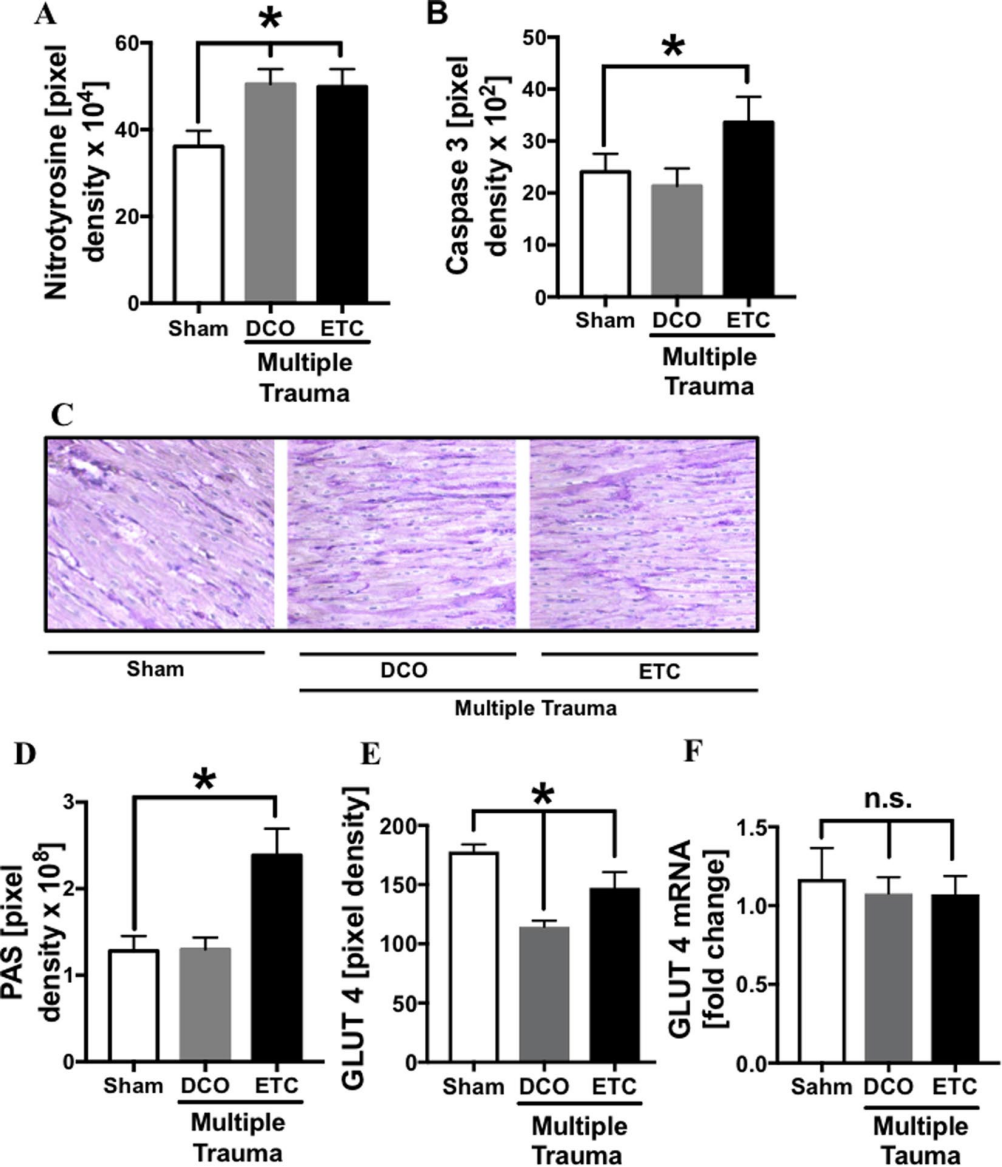
Densitometry of immunflourescence staining of nitrotyrosine in left ventricle sections from sham (white bar) treated pigs or 72 h after multiple trauma and DCO (grey bar) or ETC (black bar) treatment. (**B**) Densitomery of immuneflourescence staining of caspase 3 in left ventricular tissue sections from sham (white bar) treated pigs or 72 h after multiple trauma and DCO (grey bar) or ETC (black bar) treatment. Representative PAS staining of left ventricular tissue after sham procedure (**C**) or after multiple trauma followed by DCO or ETC treatment. (**D**) Pixel density of PAS staining in left ventricular tissue sections from sham (white bar) treated pigs or 72 h after multiple trauma and DCO (grey bar) or ETC (black bar) treatment. (**E**) Pixel density of GLUT 4 staining in left ventricular tissue sections from sham (white bar) treated pigs or 72 h after multiple trauma and DCO (grey bar) or ETC (black bar) treatment. (**F**) Expression of GLUT 4 mRNA in left ventricular tissue assessed by qRT-PCR from sham (white bar) treated pigs or 72 h after multiple trauma and DCO (grey bar) or ETC (black bar) treatment, expressed as ratio of GLUT 4 mRNA to Gapdh mRNA. *p < 0.05, n = 6 each bar.

(Figure 6) Densitry of nitrotyrosine staining revealed a minor but significantly increased expression after multiple trauma in both treatment groups compared to sham procedure (frame A). Staining of caspase 3 showed a significantly increased expression after trauma and ETC treatment (frame B) compared to sham treated animals. In sham treated animals and after multiple trauma and DCO treatment PAS staining was faint whereas after trauma and ETC treatment PAS staining was more intense (frame C), indicating increased amount of glycogen. After trauma followed by ETC and DCO treatment glucose transporter 4 (GLUT 4) was decreased on cardiomycytes compared to sham treated animals (frame D), whereas GLUT 4 mRNA was slightly but not significantly reduced after trauma compared to sham treated animals 72 h after trauma (frame E).

## Discussion

A linkage between cardiac depression after trauma and structural and molecular alterations in cardiac tissue is described in this report. During haemorrhage, cardiac oxygen need is enhanced in order to the increased cardiac effort to keep up adequate blood pressure with minimal blood volume, mirrored by increased heart rate 164/min compared to baseline heart rate 73/min (data not shown). In the present context, signs of nitrosative stress in cardiac tissue, were evident after trauma and might be linked to the cardiac impairment. High tissue concentrations of NO have been linked to cardiac depression during sepsis^22^. Moreover, in experimental sepsis and during ischemia increased glucose uptake via GLUT 4 and increased glycogen deposition in the heart were observed together with diminished cardiac performance^23,24^. In the present study, correspondingly increased deposits of glycogen in the heart after trauma and ETC treatment were observed. In contrast to findings during ischemia where GLUT 4 was found to be translocated from intracellular vesicles to the plasma membrane together with its up regulation and switch of their primary substrate from fatty acids to glucose to facilitate anaerobic gycolysis^25^ in the present study GLUT 4 on the sarcolemma of cardiomyocytes was decreased. In mice with myocardium restricted deletion of GLUT 4 during ischemia systolic and diastolic contractile dysfunction was observed^26^. Altogether this might be an attempt to explain the reversible character of cardiac dysfunction after trauma.

Besides nitrosative stress and metabolic alteration, the systemic inflammatory response to trauma and the presence of DAMPs might be important protagonists of cardiac dysfunction after trauma. The present data confirm the appearance of histones in serum early after trauma in both treatment groups and further elevation in animals with ETC treatment, indicating increased tissue damage in consequence of femoral nailing compared to external fixation. These results are in accordance with earlier studies demonstrating elevation of circulating histones after trauma^18^ and during sepsis^27^ in humans. Furthermore, a significant correlation between circulating histone levels and Sequential Organ Failure Assessment (SOFA), organ failure and lethality was observed^18^. The presence of extracellular histones in plasma was associated with accumulation in the heart^28^ *in vivo* and on the plasma membrane and the cytosol of cardiomyocytes *in vitro* ^29^ associated with impressive elevation in intracellular calcium and reactive oxygen species which has been linked to defective cardiomyocyte function^29^. In the present study synchronous time course of elevated serum histones and impaired cardiac function were observed. Correspondingly, increased extracellular histone levels after porcine multiple trauma might be linked to cardiac dysfunction. Furthermore, after multiple trauma and ETC caspase 3 in left ventricle sections was increased compared to sham treated animals. These findings are in accordance with earlier studies revealing increased apoptosis in cardiomyocytes in presence of extracellular histones *in vitro* ^29^ and cardiomyocyte apoptosis after trauma in rats^30^. Furthermore, incubation of cardiomyocytes with plasma isolated from mice after multiple trauma resulted in cardiomyocyte apoptosis *in vitro* ^31^. However, a limitation of the study is that the impact of either treatment group, femoral nailing or external fixation, on cardiac performance might be underestimated in the present model because compared to the difference of both therapy regimes in humans, where reamed femoral nailing is associated with a considerable iatrogenic trauma, in the present study femoral nailing might be less extensive and thus the difference might be less pronounced. Further studies are needed to identify direct relation between the presence of extracellular histones occurring after trauma and the appearance of cardiac dysfunction. It is possible that neutralizing of extracellular histones after trauma might represent an effective strategy for treatment of patients with cardiac depression after trauma.

Moreover, cytokines such as IL-1β and IL-6, elevated in plasma post trauma^32^, have been shown to be cardio-depressive^33,34^. In the present study, IL-1β in left ventricular homogenates was elevated after the multiple trauma impact in both treatment groups, while IL-6 was elevated after trauma and ETC. IL-6 has been shown to correlate with the severity of tissue injury in the heart^35^. Therefore, cardiac dysfunction after trauma might be provoked by increased tissue levels of cardio-depressive cytokines. Furthermore, after trauma C5aR1 protein was reduced in both therapy concepts. This finding is in contrast to earlier studies in rats showing increased C5aR1 expression on cardiomyocytes 24 h after burn injury^36^ and after cecal-ligature and puncture-induced sepsis^17^ where absence of C5aR1 was associated with improved cardiac function^37^. One possible explanation for the downregulation of C5aR1 after trauma could be an internalization of the C5aR1 triggered by C5a^38^. Of note, C5aR-C5a interaction in isolated perfused hearts caused some dysfunction of rat cardiomyocytes resulting in compromised cardiac function^17^. Cleavage of the receptor by neutrophil serine protease would be a possible explanation for reduced C5aR protein in cardiac tissue after trauma^39^ but MPO measurement in left ventricular homogenates did not indicate any increased neutrophil activity in cardiac tissue after trauma (data not shown).

In the present investigation mechanical damage, nitrosative stress and local inflammatory response after multiple trauma were associated with altered cell-to-cell and intracellular integrity. Cx43 was found to be trans-located from the intercalated disc region to the cytosol after trauma which was associated with the co-translocation of ZO-1. Gap-junction endocytosis results in disruption of functional contact between cardiomyocytes and disruption of coordinated spread of any electrical activation, which again is associated with a loss of mechanical and electrical coupling of cardiomyocytes^40^, arrhythmia and cardiac dysfunction^41^. Co-translocation of Cx43 and ZO-1 has been observed after partial or complete enzymatic dissociation of myocytes from intact ventricle associated with gap junction endocytosis^42^. Cell-cell communication through gap junctions such as Cx43 has been shown to partly prevent apoptosis *in vitro* ^43^. In the present multiple trauma study and DCO concept Cx43 was upregulated in left ventricular tissue, which was associated with absent apoptosis. Taken together cardiac alterations of Cx43 distribution and expression as well as increased apoptotic rate were observed, which to our knowledge the first time that posttraumatic cardiac tissue damage and dysfunction was linked to gap junction pathology.

Further structural cardiac proteins located in the Z-lines were investigated. α-Actinin, which is co-localized with L-type Ca^2+^ channels and stabilizes the muscle contractile apparatus in cardiomyocytes, was reduced in left ventricles after multiple trauma compared to sham treated animals. Z-disc proteins have been shown to act as responder to stretch or mechanical tension due to hemodynamic demands^44^. In the present context Z-disc proteins may be altered in response to shear forces applied to the heart during blunt chest trauma. Loss of proteins associated with the sarcomeric skeleton such as α-actinin may therefore contribute to ventricular dysfunction after trauma. Desmin, a major component of cardiac intermediate filaments, likewise located in the Z-lines, forming a physical link between nucleus, contractile proteins, mitochondria and sarcoplasmatic reticulum^45^, was up regulated after trauma and its distribution was altered compared to animals after sham procedure. Desminopathies such as desmin aggregates alter heart biomechanics and calcium dynamics^46^. Desmin was found to be increased in guinea pigs with heart failure^47^ and in mice with diastolic dysfunction^48^. In the present study desmin disorganisation in pigs after multiple trauma might be a compensatory mechanism after mechanical damage of cardiac tissue in an early reversible stage.

Taken together, our results suggest that multiple trauma in pigs leads to early cardiac dysfunction, which is associated with cardiac cell damage, local inflammation and disturbed cytoskeletal and gap junction architecture.

## Methods

This study presents partial results obtained from a large animal porcine multiple trauma model. The model has been previously described in detail by Horst *et al*.^49^.

### Animals and Anesthesia

All procedures conformed to the Society of Laboratory Animal Science (GV-SOLAS) as well as the National Animal Welfare Law and after approval by the responsible government authority (“Landesamt für Natur, Umwelt und Verbraucherschutz”: LANUV-NRW, Germany: AZ TV-No.: 84-02.04.2014.A265) and performed according to the guidelines of the Federation of European Laboratory Animal Science Association (FELASA). Eighteen 12–16 weeks old male pigs with a mean of 30 ± 5 kg body weight (Sus scrofa dormestica, Tierzucht GmbH Heinrich, Heinsberg, Germany) were included in the study. General instrumentation, anesthesia and trauma induction were described previously by Horst *et al*.^49^.

### Multiple trauma in pigs

Pigs underwent either multiple trauma including a combination (n = 12) of blunt chest trauma, penetrating liver injury and femur fracture followed by pressure-controlled haemorrhage (mean arterial pressure of 40 ± 5 mmHg, maximal withdrawal of 45% of calculated total blood volume) for 90 min or sham procedure (n = 6). For introduction of blunt chest trauma a pair of panels (steel 0.8 cm, lead 1.0 cm thickness) was placed on the right dorsal lower chest. A shock wave was induced by a bold shot (Blitz-Kerner, turbocut JOBB GmbH, Germany) which was applied onto the panel using cattle-killing cartridges (9 × 17; Dynamit Nobel AG, Troisdorf, Germany) as previously described^50,51^. Blunt chest trauma was associated with severe signs of lung contusion and rib fractures (2–3 ribs) as accessed by computer tomography^49^. Thereafter pigs were resuscitated by re-transfusion of the withdrawn blood accompanied by additional crystalloids (Sterofundin ISO^®^, 2 ml/kg body weight/h) and rewarming to normothermia (38.7–39.8 °C). Sham procedure (n = 6) included instrumentation and anaesthesia without trauma or haemorrhage. The multiple trauma group (n = 12) was randomized in two therapy arms (n = 6); external fixation of the femur fracture corresponding to damage control orthopaedics (DCO) or femoral nailing appropriate to early total care (ETC) principles.

### Transthoracic echocardiography in pigs

Echocardiograms were performed as previously described^52^. Imaging was performed according to the recommendations using a standard cardiac ultrasound machine (Vivid I©, GE Healthcare, United Kingdom). After acquisition of M-Mode images in parasternal long axis systolic and diastolic parameters were measured and shortening fraction (SF%) and ejection fraction (EF%) were calculated. The equations used were as follows:

$$EF( \% )=\frac{EDV-ESV}{EDV}\times 100$$

*(EDV* = *end-diastolic volume, ESV = end-systolic volume)*

$$SF( \% )=\frac{LVEDD-LVESD}{LVEDD}\times 100$$

*(LVEDD* = *left ventricle end-diastolic dimension, LVESD* = *left ventricle systolicdimension)*

Serial imaging was performed before, directly after, 1, 1.5, 2, 4, 5.5, 12, 24, 27, 30, 36 and 48 h after trauma.

### Sample Collection

Serum samples were collected and kept on ice before, 1.5, 3.5, 5.5, 24, 48 and 72 h after trauma. After centrifugation (2,000 g for 15 min at 4 °C) serum was removed and stored at −80 °C until analysis. Heart tissue of left ventricles was obtained 72 h after multiple trauma and fixed with 4% formalin followed by embedding in paraffin or quick-frozen in liquid nitrogen followed by storage at −80 °C until analysis.

### Histone, Troponin-I and H-FABP ELISA

Serum samples were analysed using ELISA kits according to the manufacturer’s instructions. Histones in pig serum were measured by using a Cell Death Detection ELISA^PLUS^ kit, which detects all histones (Hoffmann-La Roche, Indianapolis, IN). A histone mixture (containing H2, H2A, H2B, H3, H4) was used to establish a standard curve, as described previously^53^. Cardiac troponin I concentration in pig serum samples was measured by using ultrasensitive pig troponin-I ELISA kit (life diagnostics, West Chester, PA, USA) and heart-fatty acid binding protein by a pig cardiac fatty acid binding protein ELISA kit (life diagnostics, West Chester, PA, USA).

### IL-1β and IL-6 ELISA

Left ventricular samples were obtained 72 h after multiple trauma or sham procedure. Protein concentrations in tissue homogenates were determined by using Pierce BCA Protein Assay Kit (Thermo Fischer Scientific, Waltham, MA, USA). Porcine IL-1β and IL-6 concentrations in heart homogenates were analysed by using DuoSet® ELISA kits (R&D Systems, Wiesbaden-Nordenstadt, Germany).

### Western Blotting

Left ventricular tissue was obtained 72 h after multiple trauma or sham procedure and homogenized and lysed by using 1x RIPA Lysis Buffer (EMD Millipore) containing complete Mini-protease inhibitor and PhosSTOP protease inhibitor cocktail (Roche). Protein concentrations were determined in homogenates using Pierce® BCA Protein Assay Kit (Thermo Scientific). The samples were loaded under reducing conditions onto a 4–20% Mini-Protean®TGX™ Precast Gels (Bio-Rad Laboratories). After electrophoresis proteins were transferred by a Trans-Blot Turbo Transfer System using Mini Transfer Packs (both from Bio-Rad). The blots were blocked with 5% milk in tris-buffered saline (TBS) for 1 hour at room temperature (RT) and then incubated with antibodies (see below) overnight at 4 °C. For analysis of the pig heart homogenates rabbit anti-C5aR1 (Proteintech Group, Rosemont, IL, USA) and rabbit anti-Cx43 (Cell Signalling, Danvers, MA, USA) were used. After washing, HRP-anti-rabbit (Cell Signaling Technology, Danvers, MA, USA) was used as secondary antibody (1:15,000) at RT for 1 h followed by an additional washing step. Chemiluminescent HRP Hy Glo™ (Denville Scientific Inc, South Plainfield, NJ) was used for developing. The blots were analyzed by the ChemiDoc (BioRad Laboratories GmbH, Munich, Germany) and the Image Lab Software (Version 5.2, BioRad).

### Immunohistochemistry, H.E. and PAS staining

For immunohistochemistry formalin-fixed and paraffin embedded left ventricles were used. For C5aR1 staining polyclonal anti-CD88/C5aR1 (Acris Antibodies, Herford, Germany) was used as primary antibody. For Cx43 staining rabbit anti-pig Cx43 (Cell Signaling Technology, Danvers, MA, USA) was used. Nitrotyrosine staining was performed using rabbit anti-nitrotyrosine (Merckmillipore, Darmstadt, Germany) and caspase 3 staining by rabbit anti-cleaved caspase 3 (Cell Signaling, Danvers, MA, USA). Dako REAL^TM^ Detection System (Dako, Glostrup, Denmark) was used as secondary system. Fast hematoxylin and eosin staining-kit (Morphisto Evaluationsforschung & Anwendung GmbH, Frankfurt am Main, Germany) was used to investigate extravasal bleeding in cardiac tissue sections. PAS staining was performed using PAS-staining-kit (Merckmillipore, Darmstadt, Germany). Signal density was measured in five randomly chosen, distinct fields of vision (100x magnification) from each slide using an Axio ImagerM.1 microscope and the Zeiss AxioVision software 4.9 (Zeiss, Jena, Germany). Results are presented as mean density of each group (arbitrary units).

### Confocal Imaging

For confocal imaging formalin fixed and paraffin embedded heart tissue was used.

For Cx43 staining rabbit anti-pig Cx43 (Cell Signalling, Danvers, MA, USA) was used as primary and goat anti-rabbit (AF-488) as secondary antibody (Jackson ImmunoResearch Laboratories, West Grove, PA, USA). For zonula occludens 1 (ZO-1) staining polyclonal rabbit anti-ZO-1 (Bioss, Woburn, MA, USA) was used (AF-674). For α-actinin-2 staining rabbit anti-α-actinin (clone N1N3) (GeneTex, Ivine, CA, USA) was used as primary and goat anti-rabbit (AF-488) as secondary antibody (Jackson Immuno research Laboratories, West Grove, PA, USA). For desmin staining mouse anti-desmin (GeneTex, Ivine, CA, USA) was used as primary and goat anti-mouse (AF-647) as secondary antibody (Jackson Immuno Research Laboratories, West Grove, PA, USA). Following staining, cells were washed twice with PBS and covered with ProLong® Gold Antifade Reagent (Invitrogen).

For glucose transporter 4 (GLUT 4) staining rabbit anti-pig GLUT 4 (abcam) was used as primary and goat anti-rabbit (AF-488) as secondary antibody (Jackson ImmunoResearch Laboratories, West Grove, PA, USA). Confocal imaging was performed using Leica SP8 (Leica microsystems, Wetzlar, Germany). Evaluation of fluorescence-intensity was conducted by the Software Image J^54^.

### Detection of mRNA for Glut 4

Total RNA was isolated from pig heart homogenates by the TRIZOL® method (Thermo Fischer Scientific) according to manufacturer’s instructions. cDNA was generated and amplified (SYBR®) using reagents from Life Technologies. Amplification was performed using QuantStudio 3 (Thermo Fischer Scientific, Waltham, MA, USA). Calculation of the relative quantitative results was performed with the 2^−ΔΔCt^ algorithm. The following pig primers were used: GLUT 4 5′atgttgcggatgctatgggg 3′(fwd), GLUT 4 5′cctcgggtttcaggcacttt 3′(rev). For housekeeping gene Gapdh 5′gagtgaacggatttggccg 3′(fwd), Gapdh 5′aaggggtcattgatggcgac 3′(rev) were used.

### Statistical procedures

All values were expressed as means ± SEM. Data were analysed by one-way ANOVA followed by Dunnett’s or Tukey’s multiple comparison test. P ≤ 0.05 was considered statistically significant. GraphPad Prism 7.0 software was used for statistical analysis (GraphPad Software, Incorporated, San Diego, Ca, USA).

## References

[1] Elie MC (2006). Blunt cardiac injury. Mt Sinai J Med.

[2] Huber S (2014). Predictors of poor outcomes after significant chest trauma in multiply injured patients: a retrospective analysis from the German Trauma Registry (Trauma Register DGU(R). Scand J Trauma Resusc Emerg Med.

[3] Huber, S. *et al*. Predictors of poor outcomes after significant chest trauma in multiply injured patients: a retrospective analysis from the German Trauma Registry (Trauma Register DGU (R)). *Scand J Trauma Resus* **22**, doi:Artn 52Doi 10.1186/S13049-014-0052-4 (2014). 10.1186/s13049-014-0052-4 PMC4347585 25204466

[4] Skinner DL (2015). Blunt cardiac injury in critically ill trauma patients: a single centre experience. Injury.

[5] Crown LA, Hawkins W (1997). Commotio cordis: Clinical implications of blunt cardiac trauma. American Family Physician.

[6] Nirgiotis JG, Colon R, Sweeney MS (1990). Blunt trauma to the heart: the pathophysiology of injury. J Emerg Med.

[7] Theodoropoulos I, Cheeyandira A, Tortella BJ (2013). Traumatic Tricuspid Valve Rupture Presenting as Third-Degree Atrioventricular Block. Journal of Emergency Medicine.

[8] Eisenach JC, Nugent M, Miller FA, Mucha P (1986). Echocardiographic Evaluation of Patients with Blunt Chest Injury - Correlation with Perioperative Hypotension. Anesthesiology.

[9] Maenza RL, Seaberg D, DAmico F (1996). A meta-analysis of blunt cardiac trauma: Ending myocardial confusion. American Journal of Emergency Medicine.

[10] Rocksen D, Gryth D, Druid H, Gustavsson J, Arborelius UP (2012). Pathophysiological effects and changes in potassium, ionised calcium, glucose and haemoglobin early after severe blunt chest trauma. Injury.

[11] Meier R (2003). Effects of cardiac contusion in isolated perfused rat hearts. Shock.

[12] Kalbitz, M. *et al*. The Role of Troponin in Blunt Cardiac Injury After Multiple Trauma in Humans. *World J Surg*, doi:10.1007/s00268-016-3650-7 (2016). 10.1007/s00268-016-3650-7 27501709

[13] Manson J, Thiemermann C, Brohi K (2012). Trauma alarmins as activators of damage-induced inflammation. Brit J Surg.

[14] Lausevic Z, Lausevic M, Trbojevic-Stankovic J, Krstic S, Stojimirovic B (2008). Predicting multiple organ failure in patients with severe trauma. Can J Surg.

[15] Burk AM (2012). Early Complementopathy after Multiple Injuries in Humans. Shock.

[16] Kanse SM (2012). Factor VII-activating protease is activated in multiple trauma patients and generates anaphylatoxin C5a. Journal of Immunology.

[17] Niederbichler, A. D. *et al*. An essential role for complement C5a in the pathogenesis of septic cardiac dysfunction. *J Exp Med* **203**, 53-61, doi:jem.20051207 [pii]10.1084/jem.20051207 (2006). 10.1084/jem.20051207 PMC2118072 16380509

[18] Abrams ST (2013). Circulating histones are mediators of trauma-associated lung injury. Am J Resp Crit Care.

[19] Xu J, Zhang X, Monestier M, Esmon NL, Esmon CT (2011). Extracellular histones are mediators of death through TLR2 and TLR4 in mouse fatal liver injury. Journal of Immunology.

[20] Ferrara A, MacArthur JD, Wright HK, Modlin IM, McMillen MA (1990). Hypothermia and acidosis worsen coagulopathy in the patient requiring massive transfusion. Am J Surg.

[21] Harwood, P. J., Giannoudis, P. V., van Griensven, M., Krettek, C. & Pape, H. C. Alterations in the systemic inflammatory response after early total care and damage control procedures for femoral shaft fracture in severely injured patients. *J Trauma* **58**, 446-452; discussion 452-444 (2005). 10.1097/01.ta.0000153942.28015.77 15761335

[22] Neri M, Riezzo I, Pomara C, Schiavone S (2016). & Turillazzi, E. Oxidative-Nitrosative Stress and Myocardial Dysfunctions in Sepsis: Evidence from the Literature and Postmortem Observations. Mediators Inflamm.

[23] Levy RJ (2005). Evidence of myocardial hibernation in the septic heart. Critical Care Medicine.

[24] Levy RJ (2005). Evidence of myocardial hibernation in the septic heart. Shock.

[25] Nishino Y (2004). Ischemic preconditioning activates AMPK in a PKC-dependent manner and induces GLUT4 up-regulation in the late phase of cardioprotection. Cardiovasc Res.

[26] Sohn K (2013). Absence of glucose transporter 4 diminishes electrical activity of mouse hearts during hypoxia. Exp Physiol.

[27] Alhamdi Y (2015). Circulating Histones Are Major Mediators of Cardiac Injury in Patients With Sepsis. Critical Care Medicine.

[28] Fattahi F (2015). Organ distribution of histones after intravenous infusion of FITC histones or after sepsis. Immunol Res.

[29] Kalbitz, M. *et al*. Role of extracellular histones in the cardiomyopathy of sepsis. *Faseb J*, doi:10.1096/fj.14-268730 (2015). 10.1096/fj.14-268730 PMC4415013 25681459

[30] Jing, Z. H. *et al*. Protective Effect of Quercetin on Posttraumatic Cardiac Injury. *Sci Rep-Uk* **6**, doi:ARTN 3081210.1038/srep30812 (2016). 10.1038/srep30812 PMC4965739 27470932

[31] Tao L (2005). Mechanical traumatic injury without circulatory shock causes cardiomyocyte apoptosis: role of reactive nitrogen and reactive oxygen species. Am J Physiol-Heart C.

[32] Gebhard F, Huber-Lang M (2008). Polytrauma - pathophysiology and management principles. Langenbeck Arch Surg.

[33] Kumar A (1996). Tumor necrosis factor alpha and interleukin 1 beta are responsible for *in vitro* myocardial cell depression induced by human septic shock serum. J Exp Med.

[34] Yu XW, Liu MYG, Kennedy RH, Liu SJ (2005). Both cGMP and peroxynitrite mediate chronic interleukin-6-induced negative inotropy in adult rat ventricular myocytes. Journal of Physiology-London.

[35] Karu I (2009). Off-pump coronary surgery causes immediate release of myocardial damage markers. Asian Cardiovasc Thorac Ann.

[36] Hoesel, L. M. *et al*. C5a-blockade improves burn-induced cardiac dysfunction. *Journal of Immunology* **178**, 7902-7910, doi:178/12/7902 [pii] (2007). 10.4049/jimmunol.178.12.7902 17548628

[37] Kalbitz, M. *et al*. Complement Destabilizes Cardiomyocyte Function *In Vivo* after Polymicrobial Sepsis and *In Vitro*. *J Immunol*, doi:10.4049/jimmunol.1600091 (2016). 10.4049/jimmunol.1600091 PMC4988523 27521340

[38] Poursharifi P (2013). C5L2 and C5aR interaction in adipocytes and macrophages: Insights into adipoimmunology. Cell Signal.

[39] van den Berg CW (2014). Mechanism of Neutrophil Dysfunction: Neutrophil Serine Proteases Cleave and Inactivate the C5a Receptor. Journal of Immunology.

[40] Agullo-Pascual E, Cerrone M, Delmar M (2014). Arrhythmogenic cardiomyopathy and Brugada syndrome: Diseases of the connexome. Febs Lett.

[41] Gutstein DE (2001). Conduction slowing and sudden arrhythmic death in mice with cardiac-restricted inactivation of connexin43. Circulation Research.

[42] Barker RJ, Price RL, Gourdie RG (2002). Increased association of ZO-1 with connexin43 during remodeling of cardiac gap junctions. Circ Res.

[43] Yasui K (2000). Cell-to-cell interaction prevents cell death in cultured neonatal rat ventricular myocytes. Cardiovascular Research.

[44] Pyle WG, Solaro RJ (2004). At the crossroads of myocardial signaling - The role of Z-discs in intracellular signaling and cardiac function. Circulation Research.

[45] Capetanaki, Y. Desmin cytoskeleton: A potential regulator of muscle mitochondrial behavior and function. *Trends Cardiovas Med* **12**, 339–348, doi:Pii S1050-1738(02)00184-6, Doi 10.1016/S1050-1738(02)00184-6 (2002). 10.1016/s1050-1738(02)00184-6 12536120

[46] Ramspacher C (2015). Developmental Alterations in Heart Biomechanics and Skeletal Muscle Function in Desmin Mutants Suggest an Early Pathological Root for Desminopathies. Cell Rep.

[47] Wang XJ, Li F, Campbell SE, Gerdes AM (1999). Chronic pressure overload cardiac hypertrophy and failure in guinea pigs: II. Cytoskeletal remodeling. Journal of Molecular and Cellular Cardiology.

[48] Sheng JJ, Feng HZ, Pinto JR, Wei H, Jin JP (2016). Increases of desmin and alpha-actinin in mouse cardiac myofibrils as a response to diastolic dysfunction. J Mol Cell Cardiol.

[49] Horst K (2016). Characterization of blunt chest trauma in a long-term porcine model of severe multiple trauma. Sci Rep.

[50] Horst K (2015). Local inflammation in fracture hematoma: results from a combined trauma model in pigs. Mediators Inflamm.

[51] Eschbach D (2015). A porcine polytrauma model with two different degrees of hemorrhagic shock: outcome related to trauma within the first 48 h. Eur J Med Res.

[52] Kerut EK (2004). Technique and imaging for transthoracic echocardiography of the laboratory pig. Echocardiography.

[53] Bosmann, M. *et al*. Extracellular histones are essential effectors of C5aR- and C5L2-mediated tissue damage and inflammation in acute lung injury. *Faseb J*, doi:10.1096/fj.13-236380 (2013). 10.1096/fj.13-236380 PMC3834784 23982144

[54] Schneider CA, Rasband WS, Eliceiri KW (2012). NIH Image to ImageJ: 25 years of image analysis. Nature methods.

